# Enhancing Electrical Contact with a Commercial Polymer for Electrical Resistivity Tomography on Archaeological Sites: A Case Study

**DOI:** 10.3390/ma13215012

**Published:** 2020-11-06

**Authors:** Marco D. Vásconez-Maza, Pedro Martínez-Pagán, Hasan Aktarakçi, María C. García-Nieto, Marcos A. Martínez-Segura

**Affiliations:** 1 Department of Mining and Civil Engineering, Universidad Politécnica de Cartagena, Paseo Alfonso XIII 52, 30203 Cartagena, Spain; marco.vasconez@edu.upct.es (M.D.V.-M.); p.martinez@upct.es (P.M.-P.); cristinagarcianieto@gmail.com (M.C.G.-N.); 2 Advanced Geosciences, Europe S.L., Calle del Aire 85, 28042 Madrid, Spain; hasan@agiusa.com

**Keywords:** electrical resistivity tomography (ERT) method, polymer, carbomer, ground-electrode electrical contact enhancement, archaeology

## Abstract

This communication reports an improvement of the quality of the electrical data obtained from the application of electrical resistivity tomography method on archaeological studies. The electrical contact between ground and electrode enhances significantly by using carbomer-based gel during the electrical resistivity tomography measurements. Not only does the gel promote the conservation of the building surface under investigation, but it also virtually eliminates the necessity of conventional spike electrodes, which in many archaeological studies are inadequate or not permitted. Results evidenced an enhancement in the quality of the electrical data obtained in the order of thousands of units compared with those without using the carbomer-based gel. The potential and capabilities of this affordable gel make it appropriate to be applied to other geoelectrical studies beyond archaeological investigations. Moreover, it might solve corrosion issues on conventional spike electrodes, and electrical multicore cables usually provoked for added saltwater attempting to improve the electrical contact.

## 1. Introduction

Archaeological sites contain sensitive and fragile structures that need to be preserved and treated carefully. These sites usually have concealed constructions underneath; however, in most cases, these constructions have disappeared along with time, or only certain remains are kept in place. In the present, there are available, non-destructive exploration techniques, which are referred to as geophysical techniques, to study these invaluable and buried remains from the surface without affecting them. The geophysical assessment gives an overview of the underground, allowing identifying of subsurface geological formations, services, cavities, ancient concealed settlements, and so forth, according to a noticeable contrast of their measured physical characteristics by means of geophysics. Thus, it lets archaeologists detect buried settlement without the need of undertaking harmful operations to those protected structures such as trenching or mechanical drilling. As a result, geophysics has been extensively used in geology, mining, environmental studies, hydrogeology, agriculture, forensics, civil engineering, e.g., [1,2,3,4,5,6], and where their use in archaeology has not been different due to their proved capabilities. In fact, applied geophysics in archaeology is carried out in the early stages of a subsurface investigation, before any other invasive actions that can damage potential hidden structures occur, as a useful tool to guide and support all the whole process [7,8].

**Figure 1:**
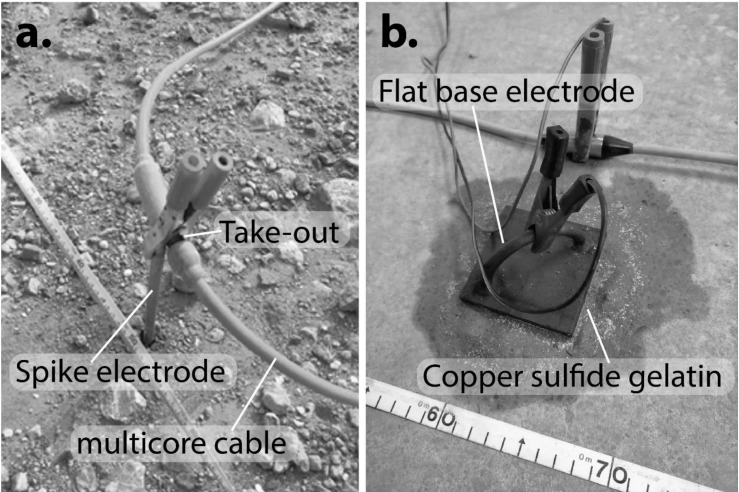
Electrodes used in electrical resistivity tomography (ERT) studies: (**a**) traditional spike electrode, and (**b**) flat-base electrode.

Among all the geophysical techniques, the electrical resistivity tomography (ERT) method is one of the most common technique used in archaeology [9,10,11,12,13] due to the fact that resistivity is a characteristic electrical property of the rock materials and it is related to their lithology, fracturing, saturation, and fluid content. Thus, by obtaining the subsurface distribution of resistivity from measurements on the surface, the structure and composition of subsurface geology, as well as its hydrology, can be inferred. The ERT method uses controlled electrical currents, which are injected into the ground, utilising electrodes to obtain electrical data from the materials underlying into the subsoil. These electrical data enable the characterisation of the underneath subsurface of a building. At each measurement, the ERT technique employs four active electrodes, from which two electrodes are used for injecting electrical current into the ground and two electrodes to measure the difference of potential. These active electrodes need to achieve good ground-electrode electrical contact to obtain quality data from the subsurface. At this point, it has to be highlighted that it is common practice to use peak electrodes (traditional spike), which are dug into the ground to both facilitate the injection of electrical current and the decreasing of ground-electrode electrical contact (Figure 1a).

However, nailing conventional electrodes into buildings of archaeological interest will likely be inadequate or not permitted in many cases (Figure 1a). This is a relevant matter of concern as this crucial drawback might make the ERT technique unpractical in many situations, especially in archaeology.

Hence, to avoid that significant limitation, as an alternative for employing ERT technique in archaeology surveys, some researchers have started using copper flat electrodes and pouring saltwater, adding bentonite, or some home-made jell [14,15] to reduce the ground-electrode electrical contact and increase the electrical flow to the subsurface (Figure 1b).

This study enhances the performance of the ERT technique by employing carbomer-based gel that virtually eliminates the need for a flat electrode.

Consequently, the aim of this communication is to report the usefulness of a new tested gel to be applied into electrical resistivity tomography (ERT) measurements in protected historical places and quantifying the electrical contact improvement between ground and electrode by means of employing this carbomer-based gel at every takeout of the multicore cable connected to the main resistivity meter.

## 2. Study Area

**Figure 2:**
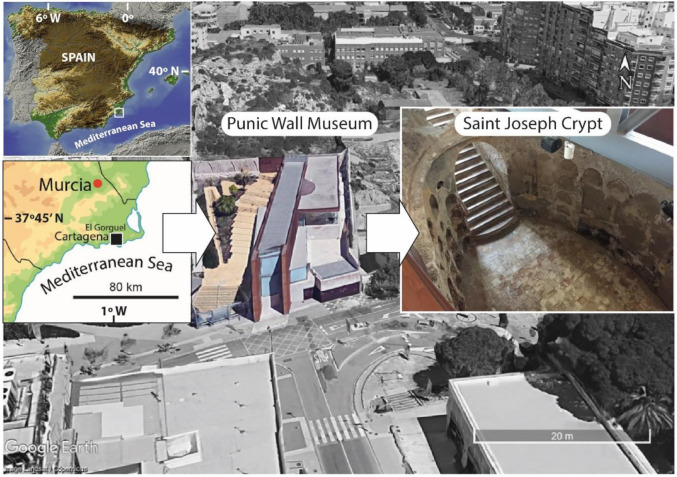
Location and view of the surveyed Saint Joseph Crypt in the Punic Wall Museum (Cartagena, Spain).

The study was carried out in the Interpretation Centre of Punic Wall (Cartagena, Spain) (Figure 2), https://puertodeculturas.cartagena.es/ficha_muralla_punica.asp?idioma=2. It is a museum built around some remarkable Punic Wall remains to preserve, study, and exhibit their structures to the visitors. The museum houses two main structures, the remains of the Carthaginian Wall from the Punic Wars, which took place in the third century (B.C.), and a funerary crypt. At its origin, the funerary crypt is believed to have belonged either to the Brotherhood of Saint Joseph or to that of Saint John of Nepomuceno, the most important Brotherhoods that existed at that time. It was built at the end of sixteenth or in the early seventeenth century [16].

Cartagena Town is in the southeast of Spain (Figure 2), which is overwhelmingly full of Roman and Carthaginian remains, because of its important role during the Punic Wars and the posterior Roman period. Most of these archaeological remains are catalogued as a fundamental cultural heritage that must be documented and protected, and their results are offered to the visitors as historical knowledge of the city foundation. Into this framework were conducted the geophysical measurements covered in this communication and their findings.

## 3. Material and Methods

### 3.1. Electrical Resistivity Tomography

The ERT investigation depth depends on the type of array employed for performing the measurements [17]. It was needed to adopt a singular procedure as the crypt of Saint Joseph is a fragile and sensitive archaeological building posing a real challenge for conventional ERT measurements. In this way, through the scientific literature, the employ of Wenner–Schlumberger array [1,2,3,18] or dipole-dipole array [4,7,19] is commonplace since they provide an excellent signal-to-noise ratio without compromising the investigation depth. Moreover, in recent indoor investigations, the employ of flat electrodes is becoming common use [20] mainly because they do not compromise the floor conditions. Thus, Zouhri et al. [21] demonstrated that flat-base electrodes are the most suitable tool for archaeological surveys. Additionally, Athanasiou et al. [22] were successful in employing flat-base electrodes to which some cellulose gel was added to improve the electrical contact.

Most of the array types use the four electrodes array principle. In general, there are two current electrodes, usually referred to as A and B, and two potential electrodes, M and N. Dipole-dipole array needs all the electrodes placed in line. Current electrodes, as well as potential electrodes, need to be close together, forming a dipole each pair. Usually, the distance between the two dipoles is at least equal or larger than dipole size.

Pole-dipole array places one current electrode as a minimum ten times the size of the study area; it is usually referred to as the “infinity”. The rest of the electrodes stay in the study area. The pair of electrodes that forms the potential dipole measures the resulting voltage [23].

**Figure 3:**
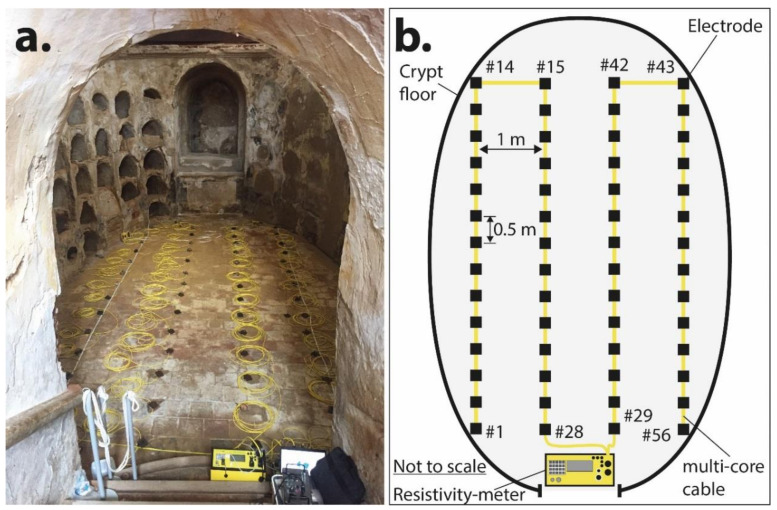
(**a**) Three-dimensional ERT layout on the Saint Joseph Crypt floor, and (**b**) sketch of the passive electrode and cable position.

Nowadays, ERT method utilizes sophisticated software and hardware that enables us to obtain a considerable amount of electrical data in a very short time. From these measured data, the subsurface structure is inferred at the interpretation stage such as geology, water content, hidden voids, archaeological remains, and so forth [24]. In this work, apparent resistivity data were recorded using a direct current resistivity multi-electrode system (AGI SuperSting R1, Advanced Geosciences Inc., Austin, Texas, USA) [25]. This system is constituted by a high-accuracy resistivity meter, a central electrode control unit, four 14-passive electrodes cables, a gas engine generator, cable connectors, and a tablet controller (Figure 3a). The four passive electrode cables allowed set up of a total of 56 passive electrodes on the crypt floor following a 3D arrangement with a total length of 27.5 m. The inter-electrode separation was 0.50 m, and the distance between parallel lines was 1 m apart (Figure 3b). It is worth noting that this resistivity multi-electrode system requires the connection to it of the cable endings next to electrode #28 and electrode #29 as it is shown in Figure 3b.

The SuperSting instrument has been used in the resistivity model for 1.2 s current injection with the range from 362 to 841 mA intervals between every two points and measuring the voltage with the range from 0.85 to 1196 mV intervals between every two other points. A total of 56 total electrodes on a 3D layout are used for a total of 516 data point measurements with dipole-dipole electrical array and 624 data point measurements with the pole-dipole array. The instrument calculates and stores a total of 1140 apparent resistivity values with the range from 1 to 95 Ω·m intervals, which were expected in such salty-water-saturated zones.

Then, all the 3D arrangement was connected to the resistivity meter. This controlled-computer central unit provided electrical measurements from the subsurface. Those measurements allowed us to generate an electrical resistivity-based 3D model from the funerary crypt’s subsurface. The quality of 3D model resistivity data is crucial to infer from them the crypt subsurface structure. As it was noted before, in this study, the solution adopted to undertake ERT measurements and evaluate their quality was to perform measurements employing two different arrays: dipole-dipole and pole-dipole array, named as measurement #1 and measurement #2, respectively.

### 3.2. Carbomer-Based Gel

As aforementioned, the study area is an important archaeological and protected site where the floor is composed of ancient and relevant tiles; therefore, any drilling or perforating on the museum floor to supply holes to install conventional spike electrodes was not allowed. What is more, the use of clay, which is commonly used with flat-base electrodes, was not allowed as it stains the protected tiles of the crypt floor. Then, a commercial gel, which is commonly used in laser epilation and/or ultrasound analyses, was tested to evaluate its actual capabilities in improving the ground-electrode electrical contact for ERT archaeological studies. The evaluation of this potential electrical contact improvement through this carbomer-based gel would be important for those archaeological sites under geophysical study where it might be expected that sensitive floor is made of significant tiles, mosaics, etc. About this commercial gel, its most numerous components are aqua, carbomer, phenoxyethanol, decylene glycol, caprly glycol, triethanolamine, and glycerine. Due to its features, it does not leave any stain, and it is removed effortlessly [6].

Carbomer is a synthetic polymer (C_3_H_4_O_2_)_n._, also known as Carbopols, which is a synthetic polymer conformed by acrylic acid chains. These several lateral chains lose protons to acquire a negative charge. Consequently, these features make it hydrophilic, allowing the absorption of water and augmenting its volume several times [26]. Due to that, it is often used in pharmaceutical applications as both a suspending agent and an emulsifying component in water-based preparations. Moreover, it has a conductivity of 0.186 S/m [27]. The gel presents an excellent thermal stability and high viscosity [7].

**Figure 4:**
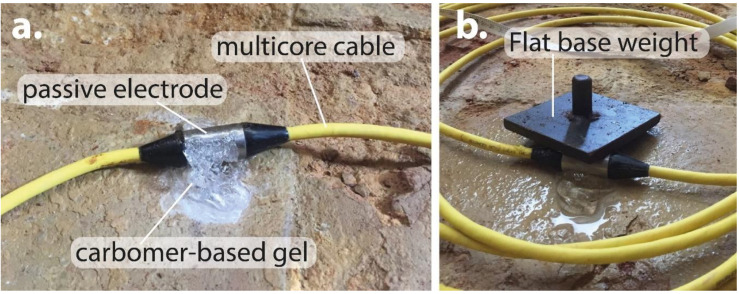
(**a**) Detail about added carbomer-based gel on the passive electrode surface, and (**b**) flat-base metal weight placed on the passive electrode.

Previously, the carbomer-based gel has been utilized to increase the electrical contact of those MRI electrodes employed in health studies [28]. Consequently, the primary aim of this study was to test and evaluate the ground-electrode contact using this carbomer-based gel. Provided that its performance is appropriate during ERT tests, it will be recommended for other similar studies. Therefore, to evaluate the ground-electrode resistance contact level, a thick layer of gel was initially added to the passive electrode surface (Figure 4a), and then some weight was placed on each electrode by means of metallic plates to further improve this contact (Figure 4b), which was enough for performing the whole measurements. After that, different electrical configurations were tested, and their results quantified, which are discussed in the next section.

## 4. Results and Discussion

Before performing the electrical resistivity tomography measurements, it is a must to check the equipment and verify if the electrical ground-electrode contact is appropriate. Otherwise, the data set would include too many anomalous data, or the measurements might not be carried out. An excessive electrical contact resistance characterized by several thousands of Ω leads to inconsistent data, making the final interpretation or characterization of the subsurface unsuitable under study. In this way, this electrical contact is the cornerstone of the entire work to obtain quality data. 

**Figure 5:**
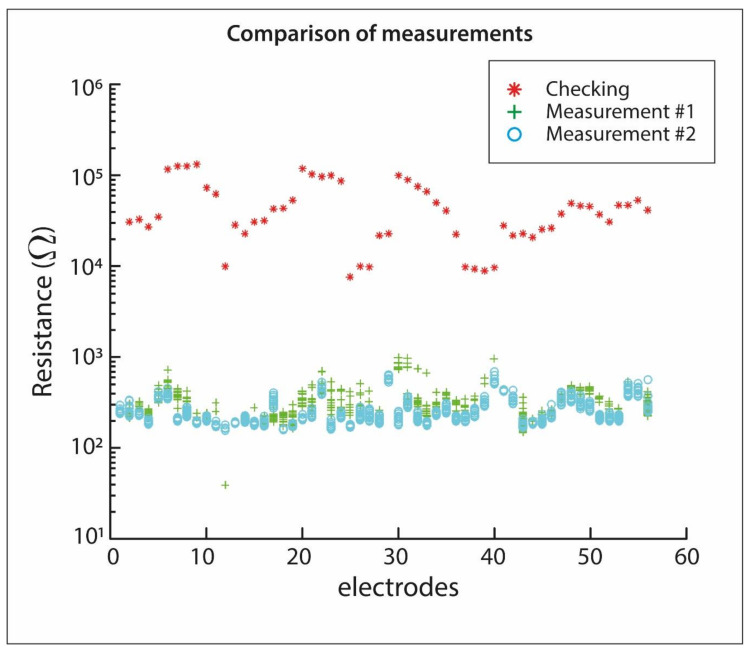
Ground-electrode resistance values at each passive electrode, in which values labelled with a red star symbol are from checking measurements, before using gel, and those values, represented by blue and cyan symbols, are from measurements #1 and #2, after using gel.

The first checking measurement consisted of putting the passive electrodes in direct contact with the crypt floor without added gel, whose values are represented by red star symbols in Figure 5. According to the scatter plot, it can be seen that the resistance values obtained during the checking measurements range from about 8000 to 140,000 Ω. Thus, this excessive resistance is considered unbearable for ERT quality data. Consequently, it was necessary to undertake new rounds of measurement tests, but this time with added carbomer-based gel. In Figure 5, the resistance contact values from this dipole-dipole (measurement #1) and from pole-dipole (measurement #2) measurements with added carbomer-based gel are represented. It can be seen in Figure 5, a noticeable improvement, in comparison with the checking measurements, of these contact resistance values, which are also represented in Figure 5 ranging from below 200 to 1000 Ω. These observed are very low contact resistance values, as a consequence of the added carbomer-base gel, which means that most of the electrical current produced from the main unit is injected into the subsurface with minimal electrical losses. Undoubtedly, the more electrical current flow is injected into the subsurface, the more accurate will be the information obtained from the subsurface structures. No matter the type of electrode utilised, the cornerstone for performing electrical resistivity tomography satisfactorily is good contact resistance.

The recommendation of contact resistance from the manufacturer of the “AGI SuperSting R1” ranges from <1800 to <5000 Ω. Achieving values under 1800 Ω is the best scenario for performing ERT technique. It is usual to achieve values under 1800 by using spike electrodes on a drillable soil [29]. Therefore, in this study, we have achieved contact resistance values under 1500 Ω by only applying carbomer-based gel, which is suitable for ERT performing.

So, the use of this gel, independently of the chosen array, dipole-dipole, or pole-dipole, enhanced considerably the electrical contact resistance, as well as did not stain the tiles of the crypt floor. It is worth noting that adding carbomer-based gel on the passive electrode surface made the employ of flat-base electrodes or other non-conventional electrodes unnecessary. However, it must be said that a little metal weight had to be placed on the passive electrodes to increase the ground-gel exchange surface. Without any doubt, the use of this commercial carbomer-based gel has turned out to be relevant to those ERT surveys that have to be undertaken indoors as well as on the tile-made floor.

**Figure 6:**
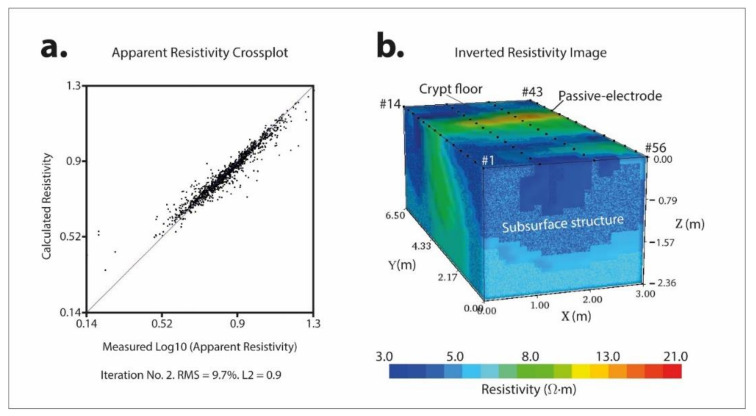
(**a**) Calculated apparent resistivity vs. measured apparent resistivity cross plot, (**b**) subsurface.

Figure 6a depicts a matching graphic where measured apparent resistivity values versus calculated apparent resistivity values have been plotted. The good match can be seen between these two sets of data that provide a root mean square (RMS) error of 9.7%, which means that the 3D subsurface inverted resistivity model (Figure 6b) is considered of high quality and accurate. In this 3D model, the spatial variation of inverted resistivity values is a consequence of the presence of inhomogeneities in the subsurface (moisture content, geology, foundations, etc.) 3D true resistivity model.

As expected, the investigation depth reached about 2.5 m. The 3D model shows significant zones with values of resistivity under 5 Ω·m that could probably be a water infiltration phenomenon. There also is a fringe with values ranging from 8 to 21 Ω·m that crosses the whole crypt horizontally. These values of resistivity show a contrast that could delimitate the wet zone from the not wet zone.

Table 1 summarises the global behaviour of the three test measurements. The minimum value of the checking is 4860% greater than the minimum value of the measurement #2, while the maximum value surpasses by 19,233% the maximum value of measurement #1. The standard deviation shows the degree of dispersion of the data. It is noticeable that after applying the carbomer-based gel, the range of resistance values is narrowed in comparison with the range obtained during the checking test. Additionally, the dispersion in the data is significantly reduced after applying the gel so the quality of the measured data will be superior to that without added gel since the data will be more stable.

## 5. Conclusions

By applying a carbomer-based gel, the electrical contact was enhanced significantly by reducing the contact resistance. This noticeable improvement was observed in the two different arrays tested: dipole-dipole (measurement #1) and pole-dipole (measurement #2), which are the common ERT arrays employed in archaeology studies. The excellent electrical contact ensures reliable performance of electrical resistivity tomography, providing accurate raw data from which the subsurface structure will be inferred. Additionally, the physical properties of the carbomer-based gel allowed the placement of the takeout directly on the crypt floor as direct contact, thus eliminating the necessity of any flat-base electrodes or other non-conventional electrodes. Due to the constituents of this carbomer-based gel, the harmful processes of corrosion observed on traditional electrodes, tweezers, and multicore-cable takeouts due to the added saltwater are eliminated or reduced. Moreover, this carbomer-based gel is affordable, which is an important point related to widespread use, and since it is a commercial cosmetic product, its access is easy and unlimited. Therefore, it can be concluded that the use of this carbomer-based gel has solved the limitations of the ERT method to be applied in archaeological sites, specifically indoor studies, where it is mandatory to improve the contact resistance without affecting the floor conditions.

## Figures and Tables

**Table 1:** Global behaviour of dataset of contact resistance.

	No Added Gel	With Added Gel	
Value	(in Ω)		
	Checking	Measurement #1	Measurement #2
**Min ^1^**	7643.3	39.4	157.3
**Max ^2^**	134,249.0	1006.1	698.0
**Med ^3^**	38,147.0	289.8	229.3
**Mean**	48,880.4	316.7	264.2
**Std ^4^**	35,243.7	134.7	97.3

^1^ Min: minimum, ^2^ Max: maximum, ^3^ Med: median, ^4^ Std: standard deviation.
